# A rapid landscape review of postpartum anaemia measurement: challenges and opportunities

**DOI:** 10.1186/s12889-023-16383-3

**Published:** 2023-07-31

**Authors:** Jennifer Yourkavitch, Hiromi Obara, Gulnoza Usmanova, Katherine E. A. Semrau, Ann-Beth Moller, Maria Nieves Garcia-Casal, Jahnavi Daru

**Affiliations:** 1 Results for Development Institute, Washington, DC USA; 2 National Center for Global Health and Medicine, Bureau of International Health Cooperation, Tokyo, Japan; 3 Jhpiego India Country Office, New Delhi, India; 4 Ariadne Labs | Brigham & Women’s Hospital and Harvard TH Chan School of Public Health, Boston, MA, USA and Harvard Medical School, Boston, MA USA; 5 Harvard Medical School, Boston, MA USA; 6 UNDP/UNFPA/ UNICEF/WHO/World Bank Special Programme of Research, Development and Research Training in Human Reproduction (HRP), World Health Organization Department of Sexual and Reproductive Health and Research, Geneva, Switzerland; 7 World Health Organization Department of Nutrition and Food Safety, Geneva, Switzerland; 8 Wolfson Institute of Population Health Science, Women’s Health Research Unit, Queen Mary University of London, London, UK

**Keywords:** Postpartum anaemia, Anaemia measurement, Women’s health

## Abstract

**Background:**

Anaemia is a reduction in haemoglobin concentration below a threshold, resulting from various factors including severe blood loss during and after childbirth. Symptoms of anaemia include fatigue and weakness, among others, affecting health and quality of life. Anaemic pregnant women have an increased risk of premature delivery, a low-birthweight infant, and postpartum depression. They are also more likely to have anaemia in the postpartum period which can lead to an ongoing condition and affect subsequent pregnancies. In 2019 nearly 37% of pregnant women globally had anaemia, and estimates suggest that 50–80% of postpartum women in low- and middle-income countries have anaemia, but currently there is no standard measurement or classification for postpartum anaemia.

**Methods:**

A rapid landscape review was conducted to identify and characterize postpartum anaemia measurement searching references within three published systematic reviews of anaemia, including studies published between 2012 and 2021. We then conducted a new search for relevant literature from February 2021 to April 2022 in EMBASE and MEDLINE using a similar search strategy as used in the published reviews.

**Results:**

In total, we identified 53 relevant studies. The timing of haemoglobin measurement ranged from within the immediate postpartum period to over 6 weeks. The thresholds used to diagnose anaemia in postpartum women varied considerably, with < 120, < 110, < 100 and < 80 g/L the most frequently reported. Other laboratory results frequently reported included ferritin and transferrin receptor. Clinical outcomes reported in 32 out of 53 studies included postpartum depression, quality of life, and fatigue. Haemoglobin measurements were performed in a laboratory, although it is unclear from the studies if venous samples and automatic analysers were used in all cases.

**Conclusions:**

This review demonstrates the need for improving postpartum anaemia measurement given the variability observed in published measures. With the high prevalence of anaemia, the relatively simple treatment for non-severe cases of iron deficiency anaemia, and its importance to public health with multi-generational effects, it is crucial to develop common measures for women in the postpartum period and promote rapid uptake and reporting.

**Supplementary Information:**

The online version contains supplementary material available at 10.1186/s12889-023-16383-3.

## Background

Anaemia is defined as a reduction in haemoglobin concentration below a threshold and can result from various factors including micronutrient deficiencies, inherited haemoglobinopathies, chronic infection, parasitic infection and blood loss [1]. According to the World Health Organization (WHO), it occurs when “the number of red blood cells…is insufficient to meet the body’s physiologic needs,” ([2]; p.1) which varies with age, gender, altitude, smoking, and stage of pregnancy. It is currently defined as having a haemoglobin concentration less than 120 g/L for non-pregnant and lactating women, and less than 110 g/L for pregnant women, adjusted for altitude and smoking [2, 3]. WHO’s thresholds to define anaemia across many populations are currently under review [4]; a recent study pooling data from multiple countries found the WHO cut-offs were higher than the 5^th^ percentile of nearly all countries, suggesting that lower cut-offs can be considered to define anaemia for children and non-pregnant women [5]. Accurate measurement is a critical component of appropriate care and understanding the global burden of morbidity [6].

Anaemia is prevalent among women and adolescent girls globally [3] and can be classified by type: micronutrient deficiencies (including iron); inflammation, aplastic, bone marrow disease, hemolytic, and sickle cell [7]. Symptoms of anaemia include fatigue, weakness, irregular heartbeats, dizziness, chest pain, and headaches, among others [7]. Anaemic women who are pregnant have an increased risk of premature delivery, a low-birthweight infant, and postpartum depression, and are more likely to have anaemia in the postpartum period [8] which can continue and affect haemoglobin levels in a subsequent pregnancy. In 2019, the global prevalence of anemia in pregnant women was nearly 37% [9]. Severe bleeding during and after childbirth can create or worsen an existing condition [10, 11]. Postpartum hemorrhage is the most common and dangerous complication of childbirth, causing 27% of maternal deaths [12] and affecting 5–10% of all births [13], contributing to postpartum anaemia. Among postpartum women in low- and middle-income countries, 50–80% are estimated to have anaemia [14].

Both postpartum hemorrhage and maternal anaemia may contribute to insufficient breastmilk supply [15, 16] with the latter also linked to a shorter duration of breastfeeding [16]. A study in Ethiopia found that 22% of lactating women were anaemic [17]; in eastern Uganda, 64% of women with a child less than one year of age were anaemic [18]. A study from South Africa found associations between iron status and depression, stress, and cognitive function among postpartum women [19]. Where anaemia is associated with breastfeeding challenges, both women’s and children’s health could be negatively affected, given the benefits that breastfeeding conveys [20].

Mild to moderate anaemia is most commonly treated orally with iron supplementation, with tablets easily dispensed during health care appointments e.g., for pre- or post-natal care, or home visits. Table 1 provides an overview of haemoglobin thresholds for anaemia severity at sea level. The prevalence of anaemia among postpartum women is currently unknown, and the condition is likely undertreated, threatening women’s and children’s health in the immediate postpartum period and long-term, including subsequent pregnancies, creating a continuous and multigenerational cycle of poor health and suboptimal growth.

**Table 1:** Haemoglobin thresholds for anaemia severity at sea level

Degree of severity	Pregnant women (g/L)	Nonpregnant women (g/L)
Mild	100–109	110–119
Moderate	70–99	80–109
Severe	< 70	< 80

*Source*: WHO [2]

The morbidity subgroup of WHO’s Mother and Newborn Information for Tracking Outcomes and Results (MoNITOR) advisory group [21] undertook this rapid landscape review of an understudied subpopulation within recent systematic reviews to produce an accelerated synthesis of evidence that identifies and characterizes postpartum anaemia measurement at three time points: the first 6 weeks, 6, and 12 months postpartum. This information informed the advisory group which was assembled for a time-limited term.

## Methods

This rapid landscape review provides information about measurement characteristics and methods for postpartum anaemia and associated outcomes, which are important for characterising common co-morbidities experienced by postpartum women with anaemia. Our rapid review follows this definition: a “knowledge synthesis in which components of the systematic review process are simplified or omitted to produce information in a timely manner” ([22]; abstract) and employs abbreviated methods compared to a systematic review, including an accelerated and targeted approach to the search and selection of references [23]. We applied the following criteria to determine study inclusion and did not record excluded sources:

- The study methods description clearly states that postpartum anaemia was identified and measured. Studies spanning the peripartum were also included provided it was clear that postpartum anaemia was identified.
- The study was published between 2012 and 2022 to ensure that the most recent and relevant data were used. We extracted any relevant biochemical and clinical measures.
- The study included women of reproductive age (between 15 and 49 years of age) in the postpartum period (up to 12 months).
- The study was published in English.
- The study involved human subjects only and was not published in the form of a letter, case report, or editorial.

We used three published systematic reviews to form the basis of the formal searches [10, 24, 25]. Using these published, peer reviewed, systematic reviews to identify relevant studies for this work provides a transparent and reproducible basis, should this work require updating. One Cochrane review [10] was designed to determine the optimum treatment strategy for postpartum anaemia. The searches for that review were carried out from database inception until April 2015 and by their nature were broad and encompassed the relevant literature for our review. Of note, within the study methods the authors clearly state that the review did not apply any restrictions on length of follow up in included studies to ensure long term benefits of harms of treatment were not excluded. Therefore, using the searches from this systematic review ensures that important studies published prior to 2015 are not missed in our review.

The second [24] was a systematic review of all the published trials in the field. Our purpose in using this one was to identify all the reported outcomes in the existing literature. This paper was the first step in the development of a minimum reporting standard for trials of iron interventions in pregnancy and the postpartum (a core outcome set), and as such, reports all the outcomes in the literature. That search strategy is described in Appendix S1 of that article [24].

The third [25], a comprehensive search of studies of anaemia in pregnancy and postpartum, was conducted from 2015 to 28th February 2021, building on other Cochrane reviews [10, 26] using the same search terms and strategies. Identifying relevant postpartum and peripartum studies from the search for that review [25] ensures that important recent studies were not missed.

**Fig. 1:**
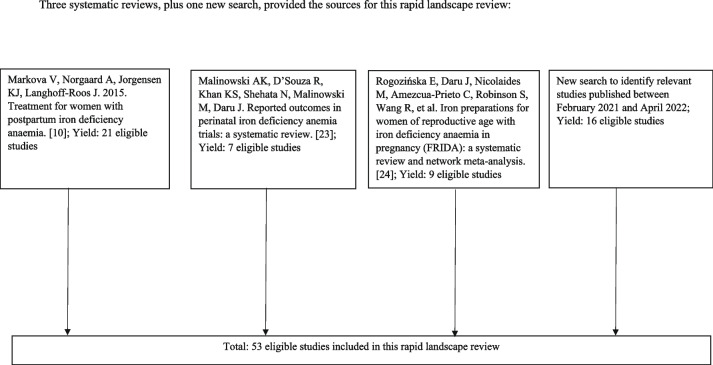
Sources of data for this rapid landscape review

We conducted a final rapid search from Feb 2021 to April 2022 in EMBASE and MEDLINE using a search strategy similar to those used by the reviews previously cited to identify any final missing relevant studies published in the last year in any language. Figure 1 illustrates the sources of data for this rapid landscape review. One experienced reviewer conducted the search and discussed the findings with other authors. Relevant data about study design, measurement methods, and other parameters were extracted to a spreadsheet, reviewed, and discussed. We included our extraction matrix as Supplemental File 1.

## Results

**Fig. 2:**
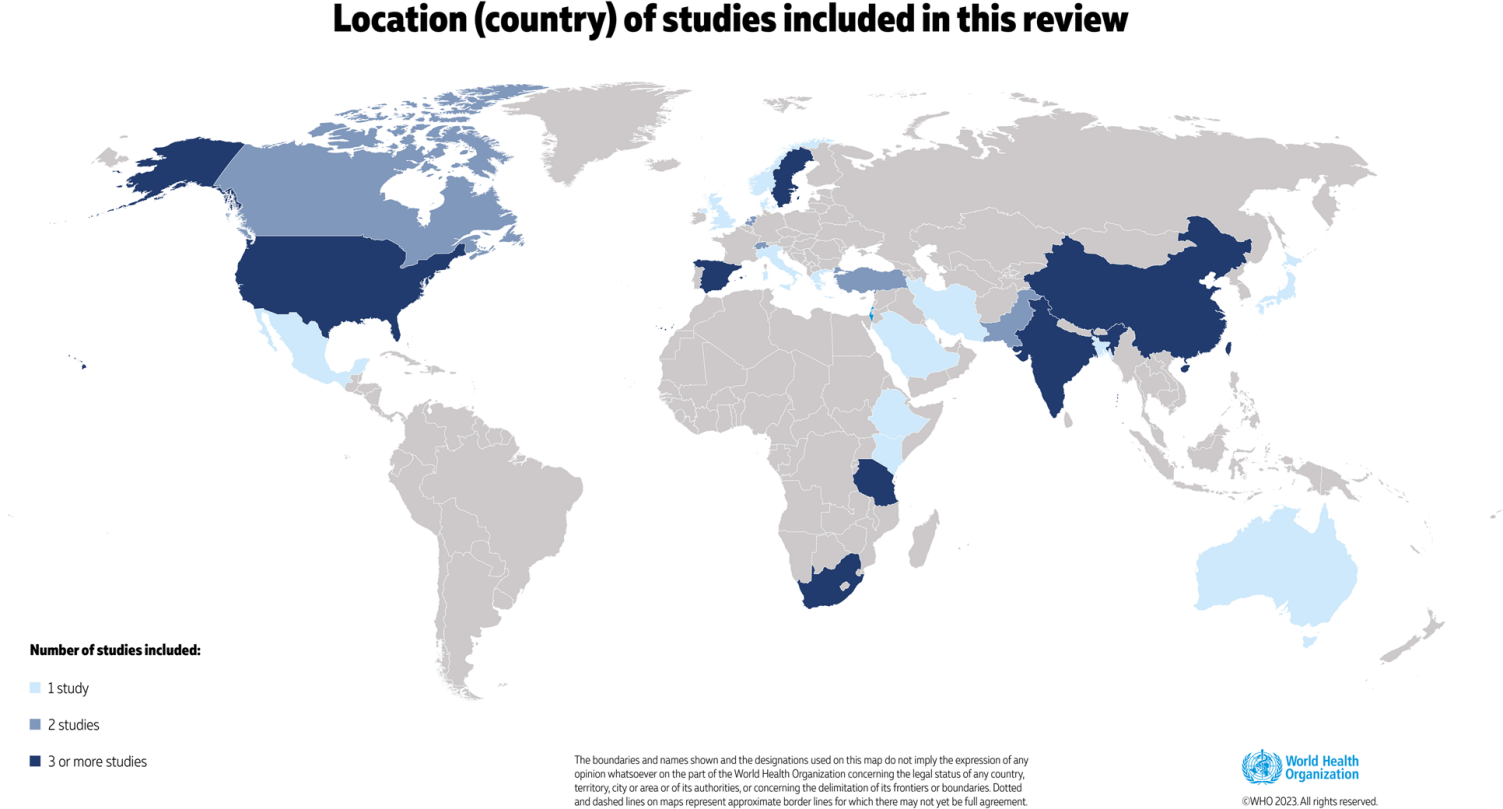
Location (country) of studies included in this review

There were 53 studies included in the review (Table 2). Of these, 27 were randomised controlled trials, 21 were prospective cohorts, and five were retrospective cohort studies. Figure 2 shows where studies were conducted, most commonly in India (8 studies) and the USA (4), Tanzania (3) South Africa (3). The majority of studies were conducted with women in the postpartum period; however, there were four studies encompassing the antenatal, intrapartum and early postnatal period. Sample sizes of the studies ranged from 31 participants to cohorts over 11,000 individuals. In most studies, the type of anaemia was not specified and only hemoglobin measurement was reported in the published data (rather than other parameters such as hematocrit, mean cell volume, etc.). There were 15 studies where participants underwent objective testing to determine if the cause of anaemia was secondary to iron deficiency, largely through studies of iron indicies. The timing of anaemia measurement ranged from the immediate postpartum period to 6 weeks after delivery; no studies measured anaemia after 6 weeks postpartum.

**Table 2:** Studies included in this review (*n* = 53)

**Authors**	**Year**	**Location**	**Study design**	**Timing of anaemia measurement (postpartum)**	**Setting of measurement**^a^	**Anaemia threshold, haemoglobin (g/L)**	**Related maternal outcomes measured**
Froessler [27]	2013	Australia	RCT^b^	72 h	Research	< 110	NR^c^
Beard [19]	2005	South Africa	RCT	6–8 weeks	Research	90 and 115	depression, cognitive ability, stress
Bhandal [28]	2006	UK	RCT	24–48 h	Research	< 90	adverse events
Daniilidis [29]	2011	Greece	RCT	NR	Research	< 80	adverse events, iron compliance
Mumtaz [30]	2011	Pakistan	RCT	24–48 h	Research	90	NR
Murray-Kolb [31]	2009	South Africa	RCT	6–8 weeks	Research	90 and 115	mother and child interactions at 10 weeks and 9 months
Perez [32]	2005	South Africa	RCT	6–8 weeks	Research	90 and 115	mother and child interactions at 10 weeks and 9 months
Guerra [33]	2012	Spain	RCT	6 weeks	Research	70 to 100	maternal mortality, infection
Jain [34]	2013	India	RCT	48 h	Research	< 80	NR
Krafft [35]	2011	Switzerland	RCT	24–48 h	Research	< 85	NR
Perello [36]	2014	Spain	RCT	48 h	Research	60–80	clinical symptoms of anaemia, depression
Prick [37]	2014	Netherlands	RCT	12–24 h	Research	48 to 79	breastfeeding
Seid [38]	2008	USA	RCT	10 days	Research	< 100	NR
Tam [39]	2005	China	RCT	2 days	Research	80 to 99	breastfeeding
Van Wyck [40]	2007	USA and Mexico	RCT	10 days	Research	less or 100	quality of life, maternal mortality, adverse events
Verma [41]	2014	India	RCT	24 h	Research	< 80	breastfeeding, length of hospital stay, blood transfusion
Wagstrom [42]	2007	Sweden	RCT	72 h	Research	< 80	infection, adverse events
Westad [43]	2008	Norway	RCT	unclear	Research	65 to 85	quality of life, adverse events
Backe [44]	2009	Sweden	RCT	48 h	Research	65 to 85	quality of life, fatigue and depression
Chaudhuri [45]	2013	India	RCT	24–48 h	Research	60–80	side effects, complications
Holm [46]	2017	Denmark	RCT	12 h	Research	> 65	change in fatigue score at 12 weeks post intervention, breastfeeding initiation, red blood cell transfusion
Hossain [47]	2013	Pakistan	RCT	24–48 h	Research	< 100	NR
Suneja [48]	2014	India	RCT	10 days	Research	70–100	NR
Breymann [49]	2007	Switzerland	Cohort	2–3 days	Research	10–12	breast milk iron stores
Dede [50]	2005	Turkey	Cohort	unclear	Research	< 90	NR
Haidar [51]	2005	Kenya	Cohort	unclear	Research	NR	NR
Mitra [52]	2012	USA	RCT	2 and 6 weeks	Research	< 120	compliance to intervention
Van Der Woude [53]	2014	Netherlands	RCT	48 h	Research	< 105	quality of life, fatigue
Zhao [54]	2019	China	Cohort	2–3 days	Research	< 120	iron in breastmilk
Kant [55]	2020	India	Cohort	48 h	Research	50–99	NR
Chandrasekaran [56]	2018	Canada	Cohort	24 h	Research	< 110	function, depression, breastfeeding rates
Kaur [57]	2021	India	Cohort	unclear	Research	50–99	NR
Yefet [58]	2020	Israel	Cohort	immediately after birth	Research	< 100	NR
Selvarajah [59]	2019	India	Cohort	6 weeks	Home	< 120	NR
Hye [60]	2022	Bangladesh	Cohort	24 h	Research	70–80	NR
Liyew [61]	2020	Ethiopia	Cohort	unclear	Research	< 120	NR
Koyuncu[62]	2017	Turkey	Cohort	unclear	Research	< 110	NR
Mremi [63]	2022	Tanzania	Cohort	6 weeks	Research	< 110	pregnancy interval
Tairo [64]	2021	Tanzania	Cohort	6 weeks	Research	< 110	breastfeeding complications
Vanobberghen [65]	2021	Tanzania	RCT	14 days	Research	< 110	quality of life, adverse events
Sharma [66]	2017	India	Cohort	unclear	Research	< 100	NR
Iancu [67]	2021	Canada	Cohort	immediately after birth	Research	< 100	prescription of oral iron on discharge, use of intravenous iron
Rubio-Álvarez [68]	2017	Spain	Cohort (retrospective)	unclear	Clinical care	< 110	NR
Garrido [69]	2017	Spain	Cohort (retrospective)	24–48 h	Clinical care	< 100	NR
Paoletti [70]	2013	Italy	RCT	unclear	Research	not defined, only measured	postnatal depression
Infante-Torres [71]	2017	Spain	Cohort (retrospective)	24 h	Clinical care	< 110	NR
Miller [72]	2016	USA	Cohort	24 h	Clinical care	< 120	postnatal depression, quality of life, fatigue
Maeda [73]	2019	Japan	Cohort	4–6 days	Research	< 100	postnatal depression
Eckerdal [74]	2016	Sweden	Cohort	at discharge from hospital	Clinical care	< 110	postnatal depression
Goshtasebi [75]	2013	Iran	Cohort	immediately after birth	Research	< 110	postnatal depression
Alharbi [76]	2014	Saudi Arabia	Cohort (retrospective)	unclear	Clinical care	< 110	postnatal depression
Armony-Sivan [77]	2012	China	Cohort	3 days	Research	< 110	postnatal depression at 6 weeks
Corwin [78]	2003	USA	Cohort (retrospective)	24 h	Home	< 120	postnatal depression, breastfeeding continuation

^a^ “Research” does not mean that automated analyzers were used or that the methods were similar or comparable

^b^Randomized controlled trial

^c^Not reported

### Specifics on anaemia measurement

All included studies reported a reduction in haemoglobin below a particular threshold as a diagnosis of postpartum anaemia. In > 90% of the studies, haemoglobin measurement was carried out within a research setting. There were six studies where haemoglobin measurement was carried out for the purposes of clinical care and subsequently reported within a published paper. Two studies measured haemoglobin in the home setting, using available point of care tests.

### Haemoglobin thresholds used for postpartum anaemia

**Fig. 3:**
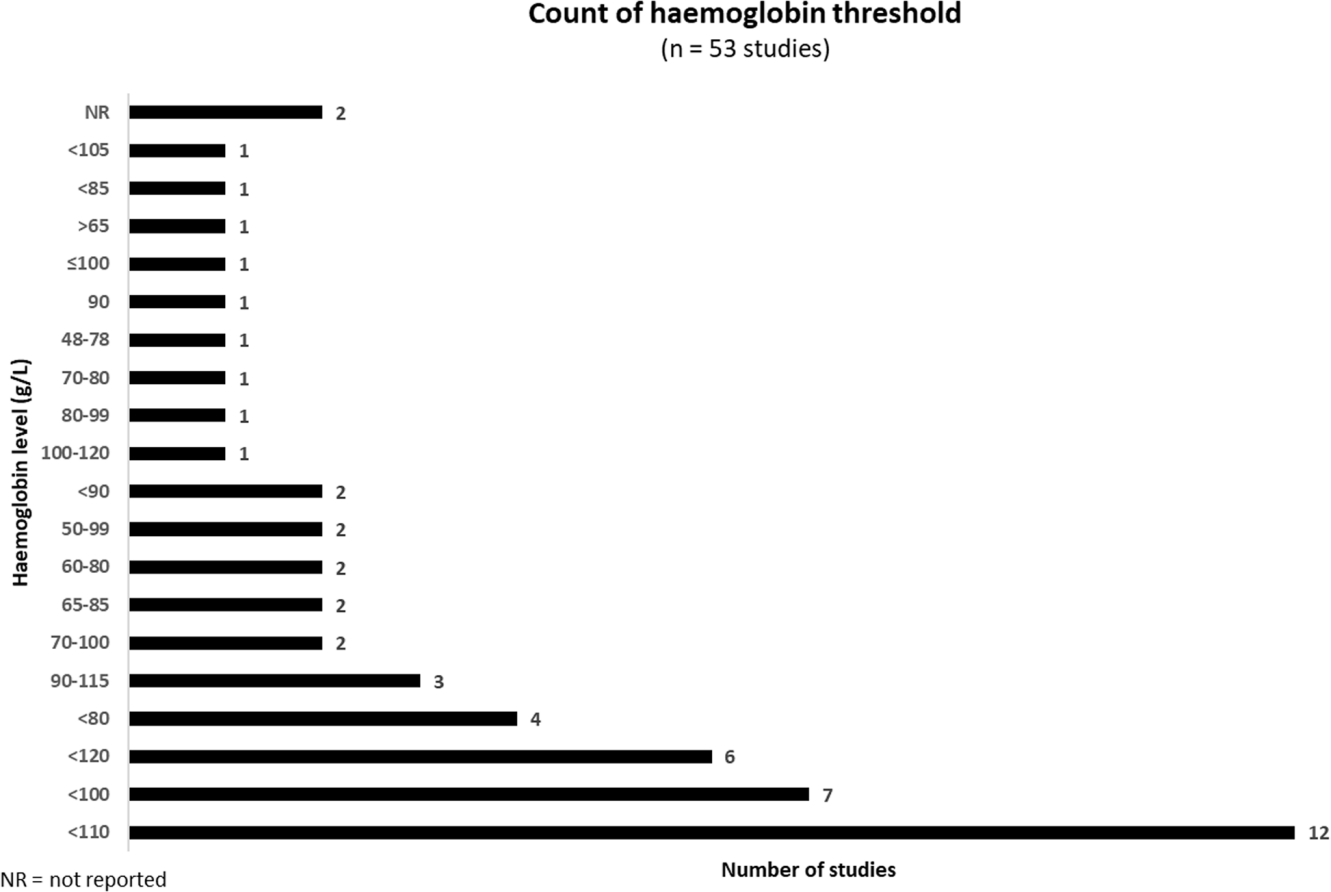
Variation in haemoglobin thresholds reported by 53 included studies

Haemoglobin thresholds used to identify anaemia in postpartum women varied considerably in the included studies (Fig. 3). Thresholds of < 120 g/L, < 110 g/L, < 100 g/L and < 80 g/L were the most commonly used. However, there were several studies that reported ranges of thresholds to demonstrate anaemia such as 50-99 g/L and 90-115 g/L to name just two ranges used.

### Measurement tools for postpartum anaemia

Haemoglobin levels (Table 2) were most commonly measured using a venous blood sample drawn and analysed within a biochemistry laboratory (36 out of 53 studies). The majority of studies provided no details on whether international standards for laboratory haemoglobin measurement were followed. Of the six studies reporting the use of point of care tests, all but one used a Haemocue® machine for such measurement. There were six studies where no specific details on the measurement tools used were provided in the published manuscripts.

There is variation in results from different measurement tools and a lack of evidence of test accuracy for alternative methods. Test accuracy studies of point of care tests compared to existing gold standards in postpartum women are unavailable. These data are needed as there is a contraction of circulating blood volume in postpartum women, therefore potentially affecting the measured values. Point of care tests compared to gold standard measurement tools require accuracy testing before they can be widely used. In addition, point of care test devices will require servicing and calibration when used.

### Maternal clinical and biochemical parameters (outcomes)

The majority of the included studies provided details on maternal biochemical parameters. For the most part, these included haemoglobin levels following iron treatment, or another intervention. Many studies also reported changes to iron indices as outcomes of iron interventions (mainly serum ferritin and transferrin receptor).

Maternal clinical parameters were less commonly reported, in only 32 out of 53 studies. Postpartum depression, quality of life and fatigue were the most commonly reported clinical parameters. For postpartum depression, the most commonly used measurement tool was the Edinburgh Postnatal Depression Score (EPDS) [79]. There was variation in the use of thresholds used from the score to identify postpartum depression. There were also reports using translated versions of the tool in settings where English was not the primary language. This occurred in three out of the eight included studies that used the EPDS. Fatigue was most commonly measured using the Multidimensional Fatigue Inventory [80]. This is used in five studies, all of which were carried out in high income settings. Quality of life was reported in four studies, using the SF-36 tool [81] to measure changes in quality of life across the postpartum period.

In addition, breastfeeding was also reported (self-reported) in six studies and provided as an event rate. Breastfeeding rates were typically measured at 6 weeks postpartum, but also at 28 days and 12 weeks postpartum in two studies.

### Infant parameters

Besides birth weight and gestational age at delivery, infant parameters were not commonly reported in the included studies. There were two studies of infant developmental milestones measured in South Africa using the Griffiths scale [82]. These two studies appeared to be linked and involved many of the same study authors.

## Discussion

Controlling the global burden of anaemia is an important public health priority: the 2025 Global Nutrition Targets pursue a 50% reduction in the prevalence of anaemia in women of reproductive age, while the 2030 Sustainable Development Goals 2 (End hunger, achieve food security and improved nutrition, and promote sustainable agriculture) and 3 (Ensure healthy lives and promote well-being for all at all ages) encompass alleviation of anaemia [83, 84]. Nutrition-specific and -sensitive interventions aimed at alleviating the burden of anaemia are advised for many low-, middle-, and high-income countries worldwide (e.g., [85]), and decisions to implement these interventions and their monitoring is founded on measuring the prevalence of anaemia and distribution in different segments of the population, notably the most vulnerable groups such as women, adolescents and children.

Monitoring postpartum health is crucial to ensure optimal health and well-being for mothers and infants [86]. This review demonstrates the need for improving postpartum anaemia measurement given the variability observed in published measures. We found that measurement of anaemia in the postpartum period varies widely, with < 120 g/L, < 110 g/L, < 100 g/L and < 80 g/L the thresholds most frequently reported. Most measurements were reported from a research, rather than a clinical- or population-based monitoring, setting, and lacked details about how haemoglobin measurement was carried out. Laboratory testing was most frequently reported as the measurement method; there were just a few reports using point-of-care tests, and the accuracy of those tests requires further study. In addition, morbidities often associated with anaemia, such as fatigue, depression, and breastfeeding difficulties, were not commonly reported by the studies in this review.

Precise and accurate diagnosis of anaemia is required to provide timely and correct treatment if needed, not only for the implications on women’s health, but also for the economic and resource implications of an incorrect diagnosis, whether false positive or false negative [4]. Haemoglobin thresholds during the postpartum period should be standardized to facilitate understanding of the true burden in local and global contexts. Next steps include incorporating measurement of postpartum anaemia in global anaemia measurement consultations such as those seeking to standardize anaemia thresholds across the lifespan, and improving or ensuring the reporting of anaemia measurement in studies. At this time, the best practice for haemoglobin determination is the use of venous blood, automated haematology analysers, and high-quality control measures. In all cases, the source, method of sample collection and method used for haemoglobin determination should be included in any report of anaemia prevalence at individual or population level. Researchers and stakeholders should be aware that data obtained using different blood sources and/or methods would not necessarily be comparable.

Anaemia during the postpartum period may have long-term health implications for the mother and her infant [11]. To increase opportunities for measurement, implementation research on point of care tests, including feasibility, availability, and acceptability is needed, along with test accuracy assessments. The measurement of clinical endpoints or morbidities associated with postpartum anaemia is needed for both women and infants. This should include measures of fatigue, weakness, breastfeeding challenges, and postpartum depression for mothers and low birthweight, preterm delivery, and breastfeeding challenges for infants.

Standard postpartum guidelines are important to help healthcare provider give adequate care and monitoring of postnatal women. Implementation of WHO guideline [87] to continue daily supplementation of oral iron and folic acid for 3 months in the postpartum period is an effective intervention in most settings. While antenatal iron supplementation is typically measured in population-based surveys, there is a missed opportunity to measure postpartum supplementation, which will provide further insight into opportunities to mitigate the burden of this morbidity. In addition, other causes of anaemia should be considered and addressed. Although iron deficiency is a major cause of postpartum anaemia [11], it is important to consider not only the reasons for the lack of iron (such as, due to significant blood loss), but also other causes, including nutrients deficiencies, and social, demographic and economic factors as either or both causes and coadjutants for anaemia [88].

One strength of this study was its efficient approach to finding relevant resources. We used previously published systematic reviews on topics that encompassed our specific question to identify articles including postpartum anaemia measurement. We then conducted a search for the recent timeframe not covered by the reviews. This is not a systematic review so there may be some missed resources; however, we believe that our methods enabled a thorough rapid report about postpartum anaemia measurement.

## Conclusions

There is marked variation in the haemoglobin thresholds used to define anaemia in women in the postpartum period. The most commonly used tool for haemoglobin measurement is a laboratory haemoglobin, with some recent use of point of care tests. The accuracy of these tests compared to the current gold standard (laboratory measure) is unknown. Clinical parameters for both women and infants are not widely reported in studies of postpartum anaemia. Anaemia and associated morbidities should be assessed, treated, and monitored to improve individual and population health. Given the high prevalence of anaemia, the relatively simple treatment for non-severe cases of iron deficiency anaemia, and its importance to public health, it is crucial to develop common measures obtained through easy methods and promote rapid uptake and reporting.

## Supplementary Information

**Additional file 1.**

## Data Availability

All data generated or analysed during this study are included in this published article.

## Abbreviations

EPDS: Edinburgh postnatal depression score
MoNITOR: Mother and Newborn Information for Tracking Outcomes and Results
NR: Not reported
RCT: Randomized controlled trial
WHO: World Health Organization
